# Efficacy and safety of abiraterone acetate plus prednisone vs. cabazitaxel as a subsequent treatment after first-line docetaxel in metastatic castration-resistant prostate cancer: results from a prospective observational study (CAPRO)

**DOI:** 10.1186/s12885-019-5974-9

**Published:** 2019-08-05

**Authors:** Javier Puente, Aranzazu González-del-Alba, Núria Sala-Gonzalez, María José Méndez-Vidal, Alvaro Pinto, Ángel Rodríguez, José Miguel Cuevas Sanz, Jacobo Rodrigo Muñoz del Toro, Eduardo Useros Rodríguez, Ángela García García-Porrero, Sergio Vázquez

**Affiliations:** 1 Medical Oncology, Hospital Clínico San Carlos. Instituto de Investigación Sanitaria del Hospital Clínico San Carlos (IdISSC), CIBERONC, C/Profesor Martín Lagos, s/n 28040 Madrid, Spain; 2 Medical Oncology, Hospital Universitario Puerta de Hierro-Majadahonda, Madrid, Spain; 3 Medical Oncology, ICO Girona, Hospital Josep Trueta, Girona, Spain; 4 Oncology Department, Maimonides Institute of Biomedical Research (IMIBIC). Reina Sofía Hospital. University of Córdoba, Cordoba, Spain; 5 Medical Oncology, University Hospital La Paz – IdiPAZ, Madrid, Spain; 6 Medical Oncology, Hospital Universitario de León, León, Spain; 7 Medical Oncology, Hospital Universitario de la Ribera, Alcira, Spain; 8 Medical Department, Janssen-Cilag S.A., Madrid, Spain; 9 Medical Oncology, Hospital Universitario Lucus Augusti, Lugo, Spain

**Keywords:** Metastatic castration-resistant prostate cancer, Abiraterone acetate, Cabazitaxel, Chemotherapy, Sequence

## Abstract

**Background:**

To describe the patterns of second-line treatment of patients with metastatic castration-resistant prostate cancer (mCRPC) after docetaxel treatment in a Spanish population, to identify the factors associated with those patterns, and to compare the efficacy and safety of the treatments most frequently administered.

**Methods:**

Observational, prospective study conducted in patients with histologically or cytologically confirmed prostate adenocarcinoma; documented metastatic castration-resistant disease; progression after first-line, docetaxel-based chemotherapy with or without other agents.

**Results:**

Of the 150 patients recruited into the study, 100 patients were prescribed abiraterone acetate plus prednisone (AAP), 44 patients received cabazitaxel plus prednisone (CP), and 6 patients received other treatments. Age (odds ratio [OR] 1.06, 95% [confidence interval] CI 1.01 to 1.11) and not elevated lactate dehydrogenase (LDH) levels (OR 0.33, 95% CI 0.14 to 0.76) were independently associated with the administration of AAP. Treatment with AAP was associated with significantly longer clinical/radiographic progression-free survival (hazard ratio [HR] 0.57, 95% CI 0.38 to 0.85) and overall survival (OS; HR 0.40, 95% CI 0.21 to 0.76) compared to CP, while no significant differences between the treatments were found regarding biochemical progression-free survival (PFS; HR 0.78 [95% CI 0.49 to 1.24]). However, in a post-hoc Cox regression analysis adjusted for potential confounders there were not differences between AAP and CP in any of the time-to-event outcomes, including overall survival. We observed no new safety signals related to either regimen.

**Conclusion:**

Second-line AAP for patients with mCRPC is the most common treatment strategy after progression with a docetaxel-based regimen. When controlling for potential confounders, patients receiving this treatment showed no differences in PFS and OS in comparison to those receiving CP, although these latter results should be confirmed in randomized controlled trials.

## Background

Since the landmark study by Huggins et al. [1] in 1941, once a patient with prostate cancer progresses after local therapy, either medical or surgical androgen-deprivation therapy constitutes the mainstay of treatment for metastatic disease [2]. In patients with metastatic castration-resistant prostate cancer (mCRPC), docetaxel in combination with prednisone was the first regimen to demonstrate an increase in survival [3] and became the standard of care as chemotherapy. Since then, several agents have been introduced for the treatment of mCRPC, making the selection of treatment more complex [4]. Among these agents, abiraterone acetate plus prednisone, enzalutamide, sipuleucel-T, radium-223 and second-line chemotherapy with cabazitaxel have demonstrated a survival benefit in patients who progressed after docetaxel therapy [5], although retreatment with docetaxel represents another therapeutic option [6]. The selection of treatment in the postdocetaxel scenario is a challenge because there are no clearly defined clinical or biological criteria for selecting the next agent, and the best sequence of treatment has not been established to date [7–9]. Thus, in a recent systematic review evaluating different treatment sequences for mCRPC, the authors only identified 16 retrospective studies and one prospective study, all of which were noncomparative studies [10]. A limited number of retrospective studies have compared the different sequences of androgen receptor-targeted therapies (ARAT) [11, 12], and no evidence is available from sequencing studies comparing ARAT vs chemotherapy prospectively.

The objective of this prospective, observational study was to describe the patterns of second-line treatment of patients with mCRPC after docetaxel treatment in a Spanish population. Secondary objectives were to identify the factors associated with those patterns and to compare the efficacy and safety of the treatments most frequently administered.

## Methods

This was an observational, prospective study conducted under real-world conditions from July 2013 to June 2016 in 24 centers in Spain. The study was reviewed and approved by the Ethics Committee of the Hospital Clínico San Carlos (Madrid, Spain). All patients provided written informed consent before being included in the study.

### Patients

Participating investigators had to include ten consecutive patients meeting the following criteria: 18 years of age or older; histologically or cytologically confirmed prostate adenocarcinoma; documented metastatic castration-resistant disease; progression after first-line, docetaxel-based chemotherapy with or without other agents; and administered a second-line treatment for mCRPC according to routine clinical practice. To be defined as castration-resistant, patients needed to have been on continuous androgen-deprivation therapy and show serum castration levels of testosterone < 50 ng/dL or < 1.7 nmol/L and three consecutive rises of prostate-specific antigen (PSA), 1 week apart, resulting in two 50% increases over the nadir, with PSA > 2 ng/mL. Patients were excluded if they exhibited cognitive deterioration that precluded understanding the patient information sheet for informed consent or if they received a second-line treatment in the setting of a clinical trial, an expanded access program or a Name Patient Program. Retreatment with docetaxel was considered a second-line treatment.

### Assessments

The study was conducted through 3 types of visits. The initial visit occurred when a patient who met the selection criteria initiated a second-line treatment after docetaxel-based, first-line treatment. Follow-up visits were scheduled every three months according to routine clinical practice. The final visit was scheduled when the patient initiated a third-line treatment, withdrew from the study or died. At the initial visit, we recorded demographic data, risk habits, medical history, and data on the first-line docetaxel-based chemotherapy, including the dates of starting and finishing first-line treatment, the number of cycles and dose, the Eastern Cooperative Oncology Group (ECOG) performance status and visual analog scale (VAS) score for pain at the beginning and end of first-line treatment, metastases at the beginning of first-line treatment, the best response to first-line treatment, the time to and type of progression in the first-line treatment, and toxicity and concomitant/palliative medication during first-line treatment. Additionally, the following information related to the second-line treatment was recorded: starting date and reason for initiating second-line treatment, therapeutic regimen, laboratory tests, ECOG performance status and VAS score for pain at the beginning of second-line treatment, and metastases at the beginning of second-line treatment. During follow-up, we recorded information on current treatment, laboratory tests, ECOG performance status, VAS score for pain, prostate cancer-related symptoms and signs, response and progression to the second-line (PSA, radiographic or clinical) based on the criteria established by each site in their routine clinical practice, concomitant/palliative medication, and toxicities. In the final visit, we recorded the vital status and the reason for finalizing treatment.

Adverse events were monitored throughout the study using open-ended questions, and the severity of these events was categorized by the investigators as mild, moderate or severe based on interference with daily life activities. Adverse events were coded with the Medical Dictionary for Regulatory Activities (MEdDRA, version 19.1).

### Statistical analysis

Sample size estimation was based on the descriptive objectives of the study. Thus, for estimating a characteristic with a relative frequency of 10% and a precision of ±4%, and assuming that 5% of patients will be excluded from the analyses due to missing data or other reasons, a total of 240 patients were required.

Efficacy outcomes included overall survival (OS), defined as the time from study inclusion (initial visit) to death from any cause, clinical or radiographic progression-free survival (PFS) and PSA progression (biochemical [b] PFS), defined according to each investigator/center criteria, and PSA response, defined as a reduction in the PSA level from baseline greater than or equal to 50%.

Quantitative variables were described using the mean and standard deviation or the median and interquartile range if required. Qualitative variables were described with absolute and relative frequencies. Binary outcomes are presented with the relative frequency and the corresponding 95% confidence interval (CI). The Kaplan-Meir method was used to describe the distribution of time-to-event outcomes, and we used the log-rank test to compare distributions among subgroups. Furthermore, time-to-event outcomes were compared using univariate Cox proportional-hazards regression analysis.

To evaluate factors associated with the prescription of abiraterone acetate plus prednisone, we used multiple logistic regression analysis with the prescription of abiraterone acetate plus prednisone as the dependent variable. Independent variables were selected from those in the bivariate analysis that were significantly different at a level of *p* < 0.2 among the following: age, ECOG performance status, diabetes, cardiovascular disorders, hypertension, symptoms, anemia (defined as Hb < 11), increased lactate dehydrogenase (LDH) (≥250 IU/L), increased alkaline phosphatase (AP) (≥160 IU/L), PSA (as a continuous variable), and early progression to first-line treatment (defined as progression during treatment). Finally, a post-hoc Cox proportional-hazards regression analyses were performed for evaluating overall survival and clinical/radiographic and biochemical progression-free survival, adjusting for those variables that were significant at a level of *p* < 0.2 in the bivariate analysis.

Statistical analyses were performed using SPSS v.22.0 (IBM Corp. Armonk, NY, USA). All tests were two-sided, and *p* < 0.05 was considered significant unless otherwise indicated.

## Results

### Demographic and clinical characteristics

We recruited 150 patients with a median age of 72.6 years at the time of initiating second-line treatment after docetaxel. All patients received docetaxel monotherapy (the median number of cycles was 6) and androgen-deprivation therapy (18 [12.0%] patients had received 3 or more hormonal manipulations). Sixty-three (42.0%) patients progressed during the first-line treatment, 31 (20.7%) within the first three months after finalizing first-line treatment and 56 (37.3%) after three months of the finalization of first-line treatment.

As second-line treatment, 100 patients were prescribed abiraterone acetate plus prednisone, and 44 patients received cabazitaxel plus prednisone. Six patients received other treatments (3 received docetaxel rechallenge; one, cisplatin; one, vinorelbine; and one, enzalutamide) and are not discussed further in this report.

The demographic and clinical characteristics of the patients are presented in (Table 1). A large number of patients showed poor prognostic factors: 46.0% had a Gleason score greater than or equal to 8, 23.5% had visceral metastases, and 79.9% had bone metastases (44% had 5 or more bone metastases). The majority of patients had an ECOG performance status ≤2. Compared with patients treated with abiraterone acetate plus prednisone, patients who received cabazitaxel plus prednisone exhibited higher PSA levels and Gleason scores, higher ECOG performance status, and a greater number of bone metastases. In contrast, patients who received abiraterone acetate plus prednisone exhibited an increased age, a greater number of visceral metastases and a higher frequency of comorbid cardiovascular disorders compared with those treated with cabazitaxel plus prednisone.

**Table 1:** Demographic and clinical characteristics

Characteristic	Second-line therapy		
	Total*	Abiraterone acetate plus prednisone	Cabazitaxel plus prednisone
Age	*N* = 149	*N* = 100	*N* = 44
Median (range), years	72.6 (49.0–89.6)	73.9 (50.9–89.6)	69.7 (51.4–84.4)
≥75 years, n (%)	62 (41.6)	47 (47.0)	13 (29.5)
Prostate-specific antigen	*N* = 147	*N* = 99	*N* = 43
Median (range), ng/ml	60.0 (0.9–3846.0)	42.8 (1.2–3222.0)	74.6 (0.9–3846.0)
Gleason score, n (%)	*N* = 139	*N* = 92	*N* = 41
Median (range)	7 (4–10)	7 (4–10)	8 (6–10)
≤6, n/N (%)	27 (19.7)	24 (26.1)	2 (4.9)
=7, n (%)	47 (34.3)	30 (32.6)	17 (41.5)
≥8, n/N (%)	63 (46.0)	38 (41.3)	22 (53.7)
ECOG performance status, n (%)	*N* = 122	*N* = 79	*N* = 40
0	31 (25.4)	22 (27.8)	7 (17.5)
1	70 (57.4)	46 (58.2)	24 (60.9)
2	20 (16.4)	10 (12.7)	9 (22.5)
3	0 (0.0)	0 (0.0)	0 (0.0)
4	1 (0.8)	1 (1.3)	0 (0.0)
Disease location, n (%)	*N* = 150	*N* = 100	*N* = 44
Bone metastases	119 (79.9)	76 (76.0)	40 (90.9)
Visceral metastases	35 (23.5)	24 (24.0)	9 (20.5)
Main current comorbidities	*N* = 150	*N* = 100	*N* = 44
Hypertension	80 (53.3)	52 (52.0)	26 (59.1)
Any cardiovascular disorder	22 (14.7)	18 (18.0)	3 (6.8)
Diabetes mellitus	26 (17.3)	17 (17.0)	8 (18.2)
Anemia	*N* = 147	*N* = 97	*N* = 44
Yes, n (%)	31 (21.1)	16 (16.5)	15 (34.1)
LDH increased	*N* = 130	*N* = 85	*N* = 39
Yes, n (%)	80 (61.5)	45 (52.9)	31 (79.5)
Alkaline phosphatase increased	*N* = 140	*N* = 93	*N* = 41
Yes, n (%)	58 (41.4)	32 (34.4)	24 (58.5)

*ECOG*, Eastern Cooperative Oncology Group; *N*, number of evaluable patients for each variable and group

*Patients receiving treatments other than abiraterone acetate or cabazitaxel are not presented, but are included in the total sample. Therefore, the total sample is not only comprised of the pool of abiraterone acetate and cabazitaxel groups

### Factors associated with the prescription of abiraterone acetate plus prednisone as a second-line treatment for mCRPC

In the bivariate analysis (Table 2), independent variables associated (*p* < 0.2) with the prescription of abiraterone acetate plus prednisone compared with receiving treatments other than abiraterone acetate included age, the presence of anemia, increased LDH, increased alkaline phosphatase (AP), and PSA level. In the multivariate analysis, age and not elevated LDH levels were associated with the administration of abiraterone acetate plus prednisone as a second-line treatment for mCRPC (Table 3).

**Table 2:** Factors associated with the prescription of second-line therapy (bivariate analysis)

Variable,	Abiraterone plus prednisone *N* = 100	Other 2nd line therapy *N* = 50	*p*-value
Age, mean (SD)	73.5 (8.3)	69.6 (8.2)	0.008
ECOG*, n (%)			
0	21 (26.6)	9 (20.5)	0.329
1	48 (60.8)	25 (56.8)	
2	9 (11.4)	10 (22.7)	
3	0 (0.0)	0 (0.0)	
4	1 (1.2)	0 (0.0)	
Lifetime diabetes (yes), n (%)	17 (17.0)	9 (18.0)	0.879
Lifetime cardiovascular disorders (yes), n (%)	19 (19.0)	8 (16.0)	0.652
Lifetime hypertension (yes), n (%)	52 (52.0)	28 (56.0)	0.643
Disease symptoms and/or signs* (yes), n (%)	57 (60.0)	34 (70.8)	0.203
Anemia (yes), n (%)	16 (16.5)	15 (30)	0.062
LDH increased (yes), n (%)	45 (52.9)	35 (77.8)	0.006
Alkaline phosphatase increased (yes), n (%)	32 (34.4)	26 (55.3)	0.018

*ECOG*, Eastern Cooperative Oncology Group; *LDH*, lactate dehydrogenase; *SD*, standard deviation

*Missing data: ECOG, abiraterone *n* = 21, other *n* = 6; Disease symptoms and/or signs, abiraterone *n* = 5, other *n* = 2; Anemia, abiraterone *n* = 3, other *n* = 0; LDH increased, abiraterone *n* = 15, other *n* = 5; Alkaline phosphatase increase, abiraterone *n* = 7, other *n* = 6 (3)

**Table 3:** Multivariate analysis of the factors associated with the prescription of abiraterone acetate plus prednisone

Factors	Beta	*p*-value	OR (95% CI)
Intercept	−2.713	0.109	
Age	0.057	0.015	1.06 (1.01 to 1.11)
LDH	−1.120	0.009	0.33 (0.14 to 0.76)

*CI*, confidence interval: *LDH*, lactate dehydrogenase; *OR*, odds ratio

### Efficacy

**Fig. 1:**
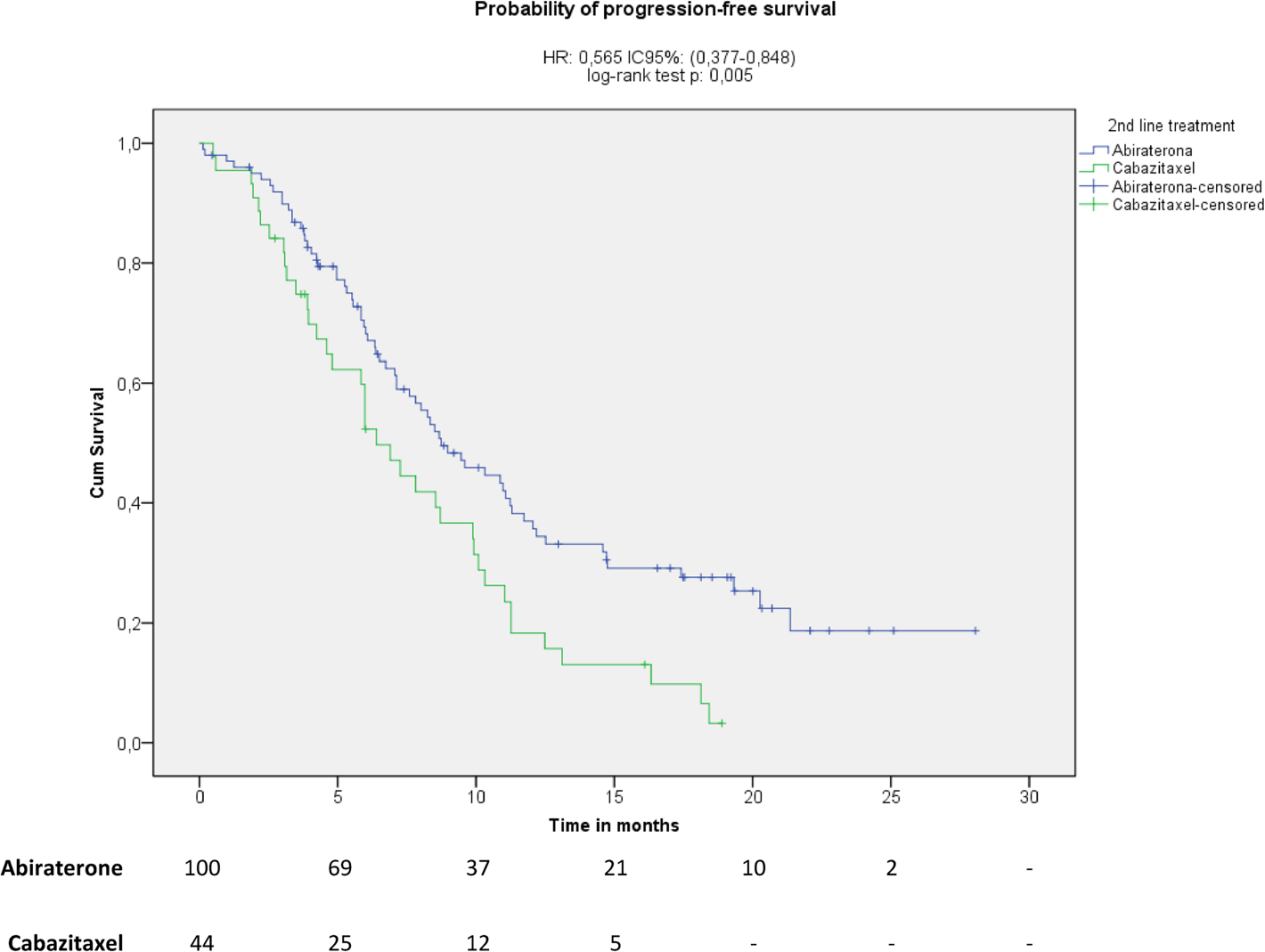
Kaplan-Meir plot for progression-free (clinical or radiological) survival

**Fig. 2:**
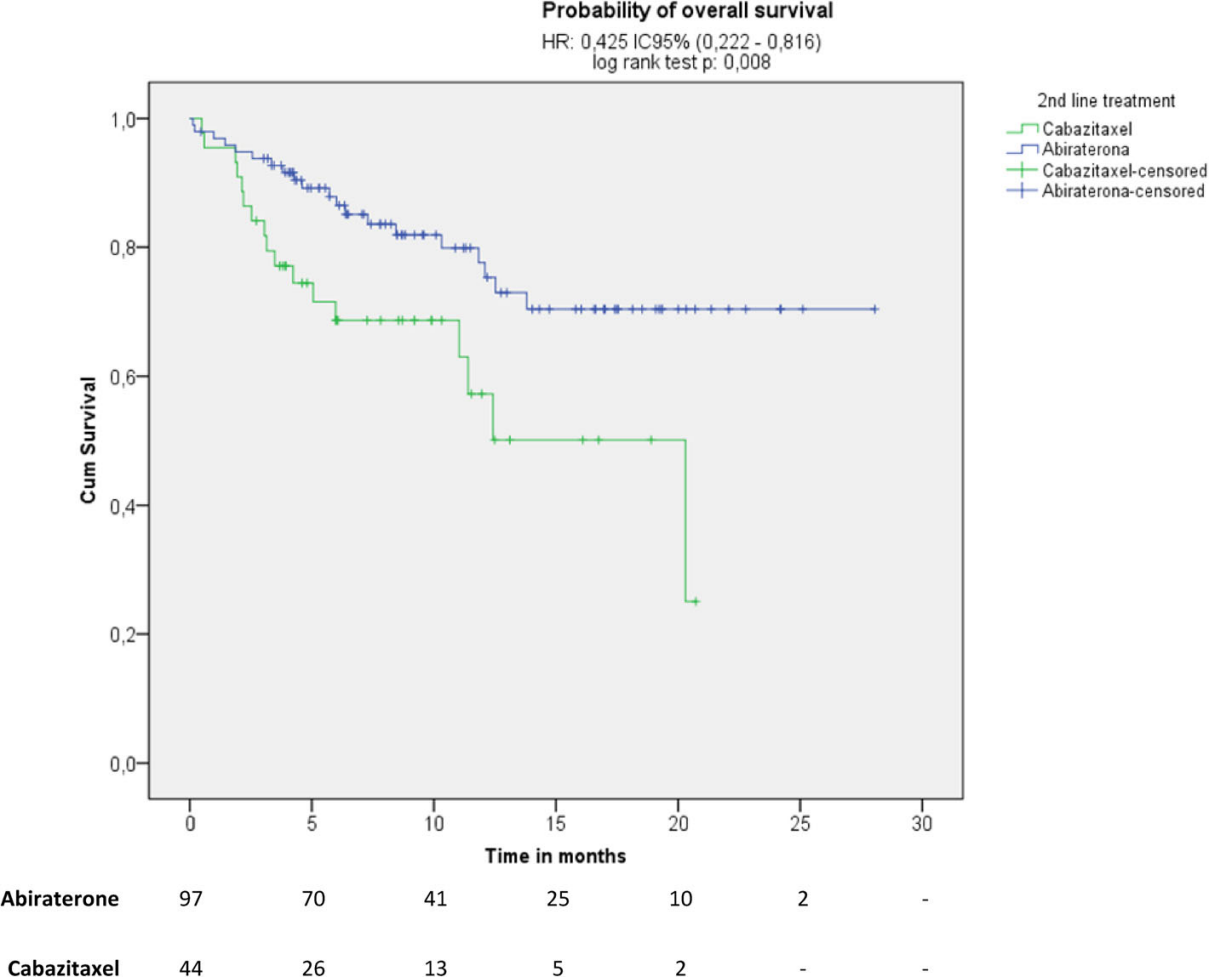
Kaplan-Meir plot for overall survival

**Fig. 3:**
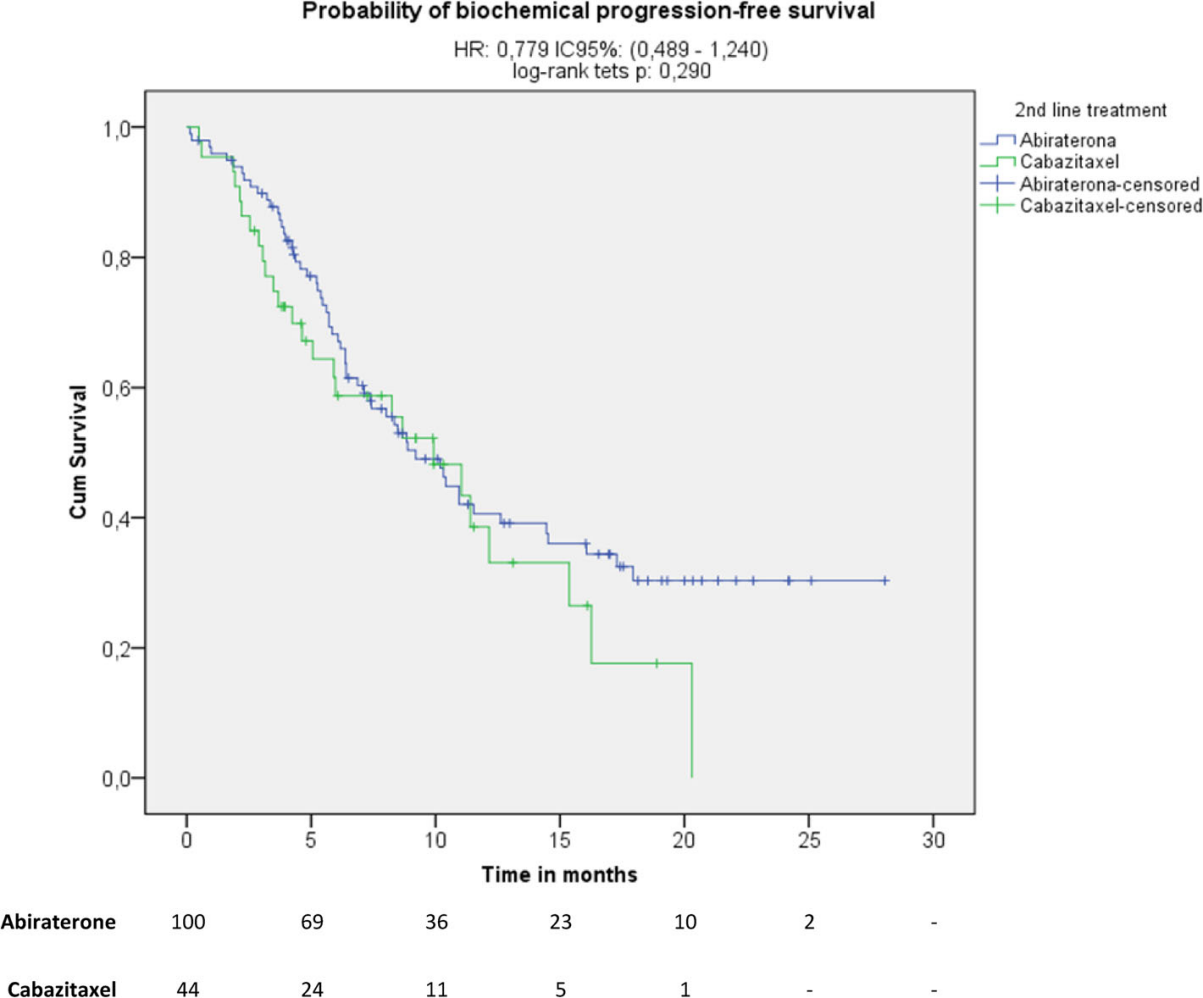
Kaplan-Meier plot for biochemical progression-free survival

Treatment with abiraterone acetate plus prednisone was associated with a 43% reduction in the likelihood of clinical/radiographic progression compared with cabazitaxel plus prednisone (Fig. 1; median 8.7 vs. 6.4 months; HR 0.57, 95% CI 0.38 to 0.85; *p* = 0.005). Similarly, the median overall survival was increased for patients treated with abiraterone acetate plus prednisone (not reached) compared with cabazitaxel plus prednisone (20.3 months) (Fig. 2; HR 0.40, 95% CI 0.21 to 0.76; *p* = 0.004). However, there was no statistically significant difference in the median biochemical PFS between both treatment groups (Fig. 3; abiraterone acetate plus prednisone: 9.2 vs. cabazitaxel plus prednisone: 9.9 months; HR 0.78 [95% CI 0.49 to 1.24]; *p* = 0.290). However, in the post-hoc Cox regression analysis adjusted for age, Gleason score, and presence of LDH increased, anemia and alkaline phosphatase increased, variables that were significant in the bivariate analyses, there were not differences between abiraterone acetate plus prednisone compared with cabazitaxel plus prednisone regarding the likelihood of clinical/radiographic progression (HR 1.12, 95% CI 0.85 to 1.49, *p* = 0.413), overall survival (HR 0.91, 95% CI 0.71 to 1.18, *p* = 0.484) or biochemical PFS (HR 1.22, 95% CI 0.91 to 1.62, *p* = 0.184). A PSA response was observed in 43 out of the 91 evaluable patients treated with abiraterone acetate plus prednisone (47.3, 95% CI 37.0 to 57.5%) and in 10 of the 31 evaluable patients treated with cabazitaxel plus prednisone (32.3, 95% CI 15.8 to 48.7%), a difference that was not statistically significant (RR 1.5, 95% CI, 0.8 to 2.6; *p* = 0.146).

### Safety and tolerability

Overall, the frequency of adverse events was increased in the cabazitaxel plus prednisone group, with the exception of pain, which was more frequent in the abiraterone acetate plus prednisone group than in the cabazitaxel plus prednisone group (28.0% vs. 20.5%). The most frequent adverse events in both treatment groups included asthenia, pain, and anemia. Additionally, an increased incidence of edema was noted among abiraterone acetate-treated patients, and an increased incidence of anorexia was noted in patients who received cabazitaxel plus prednisone (Table 4). The majority of adverse events were mild to moderate in either group. In the abiraterone acetate plus prednisone group, 302 of the 357 reported events (84.6%) were categorized as unrelated to drug treatment; the corresponding fraction for the cabazitaxel plus prednisone group was 112 of 210 (53.3%) reported events.

**Table 4:** Most frequent (≥10%) adverse events reported during treatment

	Abiraterone acetate plus prednisone (*N* = 100)				Cabazitaxel plus prednisone (*N* = 44)			
Adverse event	Mild	Moderate	Severe	Total	Mild	Moderate	Severe	Total
Asthenia	16 (16.0)	14 (14.0)	1 (1.0)	31 (31.0)	14 (31.8)	8 (18.2)	2 (4.5)	24 (54.5)
Pain	14 (14.0)	11 (11.0)	3 (3.0)	28 (28.0)	3 (6.8)	3 (6.8)	3 (6.8)	9 (20.5)
Anemia	7 7.0	7 7.0	1 (1.0)	15 (15.0)	6 (13.6)	3 (6.8)	1 (2.3)	10 (22.7)
Edema	13 (13.0)	2 (2.0)	0 (0.0)	15 (15.0)	3 (6.8)	1 (2.3)	2 (4.5)	6 (13.6)
Vomiting	7 (7.0)	0 (0.0)	2 (2.0)	9 (9.0)	4 (9.1)	1 (2.3)	0 (0.0)	5 (11.4)
Diarrhea	6 (6.0)	2 (2.0)	0 (0.0)	8 (8.0)	7 (15.9)	5 (11.4)	2 (4.5)	14 (31.8)
Anorexia	6 (6.0)	1 (1.0)	0 (0.0)	7 (7.0)	5 (11.4)	3 (6.8)	1 (2.3)	9 (20.5)
Urinary infection	1 (1.0)	5 (5.0)	1 (1.0)	7 (7.0)	1 (2.3)	3 (6.8)	2 (4.5)	6 (13.6)

## Discussion

This observational and prospective study evaluated the pattern of treatment after progression with first-line docetaxel-based chemotherapy in patients with mCRPC. Our results show that the most common therapeutic strategies after progression with first-line docetaxel-based chemotherapy in patients with mCRPC include treatment with abiraterone acetate plus prednisone in two-thirds of cases and cabazitaxel plus prednisone in one-third of cases. Similar patterns of treatment were found in a study comparing new hormonal therapies and cabazitaxel in mCRPC patients after progression on docetaxel [13]. However, it should be taken into account that by the time our study was initiated, only abiraterone and cabazitaxel were available; enzalutamide, another second-line option in patients showing progression after docetaxel, was not available. Moreover, the pattern of prescription for these two strategies differs. Compared with receiving other treatments, the likelihood of being treated with abiraterone acetate plus prednisone slightly but significantly increases with age and decreases if the patient has an increased LDH level (i.e., ≥250 IU/L), which could suggest that, in our setting, abiraterone acetate plus prednisone is preferably used as a second-line agent for patients with a lower tumor burden than those treated with other treatments (mostly cabazitaxel plus prednisone). However, despite these results, patients treated with abiraterone acetate plus prednisone also showed poor prognosis, with approximately half of the patients having a Gleason score ≥ 8 at diagnosis, one-fourth having visceral metastases, and greater than 40% of the patients having 5 or more bone metastases.

The efficacy results of abiraterone acetate plus prednisone in our study were better than those reported in the pivotal COU-AA-301 trial for this setting. In this trial [14], the median values for radiologic PFS and OS with abiraterone acetate plus prednisone were 5.6 and 15.8 months, respectively. In our study, the median time to clinical/radiographic progression was 8.7 months. After a median follow-up of 7.8 months, the median for overall survival was not reached. Furthermore, the results obtained with abiraterone acetate plus prednisone in our study were better than those reported in an observational retrospective study in Sweden [15] and in a compassionate program in Belgium [16]. Both of these studies were performed in patients with mCRPC who received chemotherapy. Similarly, the efficacy results of cabazitaxel plus prednisone were better than those reported in the previous pivotal trial. In our study, cabazitaxel plus prednisone obtained a median clinical/radiographic PFS of 6.4 months and a median OS of 20.3 months compared with 2.8 and 15.1 months [17], respectively, in the clinical trial published by de Bono in 2010. Potential explanations for these differences between our results and those from previous studies may be related to differences between treatment groups. However, it is remarkable that both treatments have good results even in real-world conditions, outside a clinical trial environment, highlighting the limitation of the applicability of pivotal trials in mCRPC to the real-world setting [18].

Prescription patterns differ between the two treatment strategies, and thus, when comparing these strategies a selection bias exists. Thus, in contrast to the crude analysis, our post-hoc Cox proportional-hazards regression analyses found no difference between abiraterone acetate plus prednisone and cabazitaxel plus prednisone in any of the time-to-event outcomes, including overall survival. These latter results are consistent with a recent meta-analysis that indirectly compared three therapies for mCRPC after docetaxel chemotherapy (abiraterone plus prednisone, enzalutamide and cabazitaxel), concluding that there was no significant differences in terms of OS favoring any of the treatments [19].

No new safety signals related to either abiraterone acetate plus prednisone or cabazitaxel plus prednisone were observed in this study, and tolerability profiles were consistent with those reported in previous trials. Moreover, both agents exhibited a low frequency of severe adverse events. However, the frequency of adverse events was increased in cabazitaxel plus prednisone patients. In addition, a higher proportion of adverse events was related to the study drug among cabazitaxel plus prednisone-treated patients than among patients who received abiraterone acetate plus prednisone. These results suggest that abiraterone acetate plus prednisone could be better tolerated than cabazitaxel plus prednisone in this setting.

In addition to the lack of balance between abiraterone acetate plus prednisone and cabazitaxel plus prednisone groups at the initial visit, our study has some limitations that should be noted. In addition to the currently available enzalutamide in Europe, abiraterone acetate plus prednisone has been demonstrated to be effective as a first-line treatment in chemotherapy-naïve patients with mCRPC [20, 21], and this indication has already been granted in Europe. Therefore, it is very likely that the second-line setting for mCRPC has changed with respect to the setting described in this study. For instance, current data suggest that the sequence of abiraterone acetate plus prednisone followed by docetaxel would be common in this new scenario [22]. A major limitation of the study is that, due to the non-interventional nature of the study, evaluations were not the standard ones used in clinical trials; for instance, adverse events were not graded using the Common Terminology Criteria for Adverse Events. We did not reach the sample size initially estimated and thus random error is increased, which constitutes another major limitation of our study.

## Conclusions

Overall, our results indicate that second-line abiraterone acetate plus prednisone for patients with mCRPC is the most common treatment strategy after progression with a docetaxel-based regimen. In this real-world setting, abiraterone acetate plus prednisone is a useful, valid treatment for mCRPC after docetaxel, and does not differ from cabazitaxel plus prednisone in terms of PFS and OS. These latter results should be confirmed in randomized controlled trials, preferably with a pragmatic design, which will also help to elucidate whether efficacy results with abiraterone acetate plus prednisone or cabazitaxel plus prednisone are superior to those reported in the pivotal explanatory trials.

## Data Availability

The data that support the findings of this study are available from Janssen Spain but restrictions apply to the availability of these data, which were used under license for the current study, and so are not publicly available. Data are however available from the authors upon reasonable request and with permission of Janssen Spain.

## Abbreviations

AAP: Abiraterone acetate plus prednisone
AP: Alkaline phosphatase
ARAT: Androgen receptor-targeted therapies
bPFS biochemical: biochemical progression-free survival
CP: Cabazitaxel plus prednisone
ECOG: Eastern Cooperative Oncology Group
mCRPC: Metastatic castration-resistant prostate cancer
MEdDRA: Medical Dictionary for Regulatory Activities
OR: Odds ratio
OS: Overall survival
PFS: Progression-free survival
PSA: Prostate-specific antigen
VAS: Visual analog scale

## References

[1] Huggins C, Hodges CV (1941). Studies on prostatic cancer. I. the effect of castration, of estrogen and of androgen injection on serum phosphatases in metastatic carcinoma of the prostate. Cancer Res.

[2] Attard G, Parker C, Eeles RA, Schroder F, Tomlins SA, Tannock I (2016). Prostate cancer. Lancet..

[3] Tannock IF, de Wit R, Berry WR, Horti J, Pluzanska A, Chi KN (2004). Docetaxel plus prednisone or mitoxantrone plus prednisone for advanced prostate cancer. N Engl J Med.

[4] Cookson MS, Lowrance WT, Murad MH, Kibel AS (2015). Castration-resistant prostate cancer: AUA guideline amendment. J Urol.

[5] Ritch CR, Cookson MS (2016). Advances in the management of castration resistant prostate cancer. BMJ..

[6] Bracarda S, Caserta C, Galli L, Carlini P, Pastina I, Sisani M (2015). Docetaxel rechallenge in metastatic castration-resistant prostate cancer: any place in the modern treatment scenario? An intention to treat evaluation. Future Oncol.

[7] Climent MA, Leon-Mateos L, Del Alba AG, Perez-Valderrama B, Mendez-Vidal MJ, Mellado B (2015). Updated recommendations from the Spanish oncology genitourinary group for the treatment of patients with metastatic castration-resistant prostate cancer. Crit Rev Oncol Hematol.

[8] Crawford ED, Petrylak DP, Shore N, Saad F, Slovin SF, Vogelzang NJ (2017). The role of therapeutic layering in optimizing treatment for patients with castration-resistant prostate cancer (prostate Cancer radiographic assessments for detection of advanced recurrence II). Urology..

[9] Cassinello J, Arranz JA, Piulats JM, Sanchez A, Perez-Valderrama B, Mellado B (2018). SEOM clinical guidelines for the treatment of metastatic prostate cancer (2017). Clin Transl Oncol.

[10] Lebdai S, Basset V, Branchereau J, de la Taille A, Flamand V, Lebret T (2016). What do we know about treatment sequencing of abiraterone, enzalutamide, and chemotherapy in metastatic castration-resistant prostate cancer?. World J Urol.

[11] Miyake H, Hara T, Tamura K, Sugiyama T, Furuse H, Ozono S (2016). Comparative assessment of efficacies between 2 alternative therapeutic sequences with novel androgen receptor-axis-targeted agents in patients with chemotherapy-naive metastatic castration-resistant prostate cancer. Clin Genitourin Cancer.

[12] Maughan BL, Luber B, Nadal R, Antonarakis ES (2017). Comparing sequencing of abiraterone and enzalutamide in men with metastatic castration-resistant prostate cancer: a retrospective study. Prostate..

[13] Oh WK, Miao R, Vekeman F, Sung J, Cheng WY, Gauthier-Loiselle M (2017). Patient characteristics and overall survival in patients with post-docetaxel metastatic castration-resistant prostate cancer in the community setting. Med Oncol.

[14] Fizazi K, Scher HI, Molina A, Logothetis CJ, Chi KN, Jones RJ (2012). Abiraterone acetate for treatment of metastatic castration-resistant prostate cancer: final overall survival analysis of the COU-AA-301 randomised, double-blind, placebo-controlled phase 3 study. Lancet Oncol.

[15] Svensson J, Andersson E, Persson U, Edekling T, Ovanfors A, Ahlgren G (2016). Value of treatment in clinical trials versus the real world: the case of abiraterone acetate (Zytiga) for postchemotherapy metastatic castration-resistant prostate cancer patients in Sweden. Scand J Urol.

[16] van Praet C, Rottey S, van Hende F, Pelgrims G, Demey W, van Aelst F (2016). Abiraterone acetate post-docetaxel for metastatic castration-resistant prostate cancer in the Belgian compassionate use program. Urol Oncol.

[17] de Bono JS, Oudard S, Ozguroglu M, Hansen S, Machiels JP, Kocak I (2010). Prednisone plus cabazitaxel or mitoxantrone for metastatic castration-resistant prostate cancer progressing after docetaxel treatment: a randomised open-label trial. Lancet..

[18] Westgeest HM, Uyl-de Groot CA, van Moorselaar RJA, de Wit R, van den Bergh ACM, Coenen J (2016). Differences in trial and real-world populations in the dutch castration-resistant prostate cancer registry. Eur Urol Focus.

[19] Fryzek JP, Reichert H, Summers N, Townes L, Deuson R, Alexander DD (2018). Indirect treatment comparison of cabazitaxel for patients with metastatic castrate-resistant prostate cancer who have been previously treated with a docetaxel-containing regimen. PLoS One.

[20] Ryan CJ, Smith MR, de Bono JS, Molina A, Logothetis CJ, de Souza P (2013). Abiraterone in metastatic prostate cancer without previous chemotherapy. N Engl J Med.

[21] Ryan CJ, Smith MR, Fizazi K, Saad F, Mulders PF, Sternberg CN (2015). Abiraterone acetate plus prednisone versus placebo plus prednisone in chemotherapy-naive men with metastatic castration-resistant prostate cancer (COU-AA-302): final overall survival analysis of a randomised, double-blind, placebo-controlled phase 3 study. Lancet Oncol.

[22] de Bono JS, Smith MR, Saad F, Rathkopf DE, Mulders PF, Small EJ (2017). Subsequent chemotherapy and treatment patterns after abiraterone acetate in patients with metastatic castration-resistant prostate cancer: post hoc analysis of COU-AA-302. Eur Urol.

